# Synthesis and Analgesic Activity of η^6^-(Anisole)- Triscarbonyl-Chromium(0)

**DOI:** 10.1155/MBD.1999.25

**Published:** 1999

**Authors:** Márcio Luís Andrade e Silva, Alberto Federman Neto, Joseph Miller

**Affiliations:** 1 Universidade de Franca Dept. of Chemistry Avenida Dr. Armando Salles Oliveira 201, Campus Franca SP 14404-600 Brazil; 2 Universidade de São Paulo Faco De Filosofia Ciências e Letras de Ribeirão Preto Dept. of Chemistry, Campus Ribeirão Preto SP 14040-901 Brazil; 3 Universidade de São. Paulo Fac. De Ciências Farmacêutcas de Ribeirão Preto Campus Ribeirão Preto SP 14040-903 Brazil; 4 Universidade Federal da Paraíba Laboratório de Tecnologia Farmacêutica P. O Box - 5009 João Pessoa PB 58051-970 Brazil

## Abstract

The general method for synthesis the η^6^-(arene)-triscarbonyl-chromium(0) complexes was modified and applied for preparation of η^6^-(anisole)-triscarbonyl-chromium(0) and the study of its analgesic activity was undertaken. A significant analgesic activity was observed after intraperitoneal injection, in Wistar rats. Two doses (30 and 50 mg/Kg of the body weight) of η^6^-(anisole)- triscarbonyl-chromium(0) were injected and the analgesic activity was evaluated by the Hot Plate Test method. They showed a significant analgesic effect in comparison with the control group and the group treated with dipyrone standard, but not so high when compared with the group treated with morphine standard. Overall, it was observed that the η^6^-(anisole)- triscarbonyl-chromium(0) was easily obtained by the modified synthetic method and was effective in increasing the pain threshold.


### SYNTHESIS AND ANALGESIC ACTIVITY OF $\eta^6$-(ANISOLE)-TRISCARBONYL-CHROMIUM(0)

Márcio Luís Andrade e Silva[1,2], Alberto Federman Neto*[2,3] Joseph Miller[4]

[1] Universidade de Franca, Dept. of Chemistry, Avenida Dr. Armando Salles Oliveira, 201, Campus, 14404-600 - Franca - SP - Brazil

[2] Universidade de São Paulo, Fac. De Filosofia, Ciências e Letras de Ribeirão Preto, Dept. of Chemistry, Campus,14040-901 - Ribeirão Preto - SP- Brazil

[3] Universidade de São Paulo, Fac. De Ciências Farmacêuticas de Ribeirão Preto, Campus, 14040 - 903 - Ribeirão Preto - SP - Brazil

[4] Universidade Federal da Paraíba, Laboratório de Tecnologia Farmacêutica, P. O . Box - 5009, 58051-970 João Pessoa - PB - Brazil

### Abstract

The general method for synthesis the $\eta^6$-(arene)-triscarbonyl-chromium(0) complexes was modified and applied for preparation of $\eta^6$-(anisole)-triscarbonyl-chromium(0) and the study of its analgesic activity was undertaken. A significant analgesic activity was observed after intraperitoneal injection, in Wistar rats. Two doses (30 and 50 mg/Kg of the body weight) of $\eta^6$-(anisole)- triscarbonyl-chromium(0) were injected and the analgesic activity was evaluated by the Hot Plate Test method. They showed a significant analgesic effect in comparison with the control group and the group treated with dipyrone standard, but not so high when compared with the group treated with morphine standard. Overall, it was observed that the $\eta^6$-(anisole)- triscarbonyl-chromium(0) was easily obtained by the modified synthetic method and was effective in increasing the pain threshold.

### Introduction

Arene triscarbonyl chromium complexes have been known since the fifties and have been extensively studied[1,6]. The most common method for their preparation is based on the reaction between hexacarbonyl chromium and arenes[2]. Nevertheless, various problems are encountered. Some organometallic chromium reagent is lost from the reaction medium by sublimation[3], while its low level of reactivity requires the use of high boiling point solvents and/or long reaction times[2-5].

Toma and his co-workers observed that yields are low, due to incomplete reaction, when low boiling point solvents are used. However, when high boiling point solvents are used, yields are also reduced due to decomposition[4]. Many solvents may be used: they include excess of arene , dioxane, acetone, esters, alkanes, amines, ethers, nitriles etc[5-7]. Classical, but now obsolete techniques employed autoclaves[1], sealed vessels[6] etc. and special glassware (Strohmeir apparatus)[8,11].

The most used[9] procedure is that of Mahaffy and Pauson[3] with dibutyl ether/THF as solvent, under reflux. Toma and his co-workers modified this method using decalin/butyl acetate[4,18], dibutyl ether/butyl acetate[18], pure decalin[2], pure butyl acetate, or alkyl formates as solvent[5,7].

To the best of our knowledge, neither $\eta^6$-(anisole)- triscarbonyl-chromium(0) nor any other organometallic compound have been screened for analgesic activity.

It is convenient at this point to make some general on analgesic activity.

The pain is part of a defensive reaction against dysfunction of the organism or imbalance in its functions, as well as against potentially dangerous stimulus.[13] Analgesia by increase of the pain threshold may be induced by electrical stimulation[12], or, more easily, by drugs.

There are many drugs to reduce pain and a few have been used for centuries. They include morphine, dipyrone[20], aspirin and other salicylates[21-25]. However, we are not aware of any reports on the use of organo-metallic compounds as analgesics.

The present work reports a modified method for synthesis of $\eta^6$-(arene)-triscarbonyl-chromium(0)complexes, represented by the preparation of $\eta^6$-(anisole)-triscarbonyl-chromium(0), together with a report on it's analgesic activity.

**Materials and Methods**

Experimental

Synthesis of $\eta^6$-(Arene)-Triscarbonyl Chromium(0) complexes:

Chromium hexacarbonyl, $Cr(CO)_6$ (Aldrich) was purified by dissolution in hexane, filtration through an alumina (Brockmann, activity I) column and evaporation of the solvent in a stream of nitrogen.

Ethyl acetate (reagent), butyl acetate (Aldrich), dibutyl ether (Aldrich) were refluxed for two hours over anhydrous potassium carbonate (to remove acidic impurities), then twice distilled.

Representative Preparation: $\eta^6$-(Anisole)-triscarbonyl chromium(0)

Chromium hexacarbonyl (1.1 g, 5 mmol) and anisole (0.7g, 7 mmol) was dissolved in 5 mL of dibutyl ether and 1 mL of butyl acetate or ethyl acetate was added. The mixture was protected from light and deaerated by bubbling with a very slow stream of $N_2$ and then refluxed using a long Liebig condenser. The sublimed $Cr(CO)_6$ was returned to the flask with a glass rod, and the bubbling of the $N_2$ continued during all the reflux period, for easily removal of the formed carbon monoxide.

The reflux was continued until no more sublimate appeared at the drop of the condenser (5-6 hours). After this time, the mixture was cooled and the excess of reagent and solvents were removed at 40 - 60$^0$ under vacuum, and the residue dissolved in acetone, filtered through silica (Merck, 60) in a small column, then eluted with acetone. Acetone was partially removed under vacuum. Hexane was added, and the mixture cooled in freezer during 12 hours. The precipitate $\eta^6$-(anisole)-triscarbonyl chromium(0) was isolated in the form of air stable yellow crystals, m.p. 83 $^0$C (literature, 84 - 85 $^0$C[3]). Yields: 74 % (using ethyl acetate) and 86 % (using butyl acetate). With the same procedure, some other $\eta^6$-(Arene)-Triscarbonyl Chromium(0) complexes were prepared (see Results and Discussion)

Analgesic Activity Evaluation Method:

Animals

Male Wistar rats were used, weighing 170g approximately and divided into groups of five animals. The animals were supplied from the Central for Animal Care of University of Franca. The animals were maintained in cages, with water and feeding "*ad libitum.*"

Algesimetric Test : Hot - Plate

The hot plate method, modified for rats was used[16] This method utilises an aluminium plate maintained at 51±1$^0$C, by means of water from a thermostatic water bath . An acrylic box measuring 24 x 18 x 18 cm was placed on the plate to impede the escape of animals.

The act of licking the hind paws was observed as a response, registering the elapsed time from the moment that the animal was placed on the hot plate until the response was obtained[17] (latency time).

To avoid lesions in the animals paws and consequently tissue damage which could affect subsequent readings in the absence of stimuli, the animals were removed from the plate after 30 s exposure.

Procedure for Measurement:

Before any treatment was given, the latency time was measured in three successive tests, carried out at intervals of 10 minutes. The average of the three measurements was considered as being the "basal latency" value. After intraperitoneal administration, the test latency times were re-measured at time intervals of 10 in 10 minutes, for 40 minutes.

The effects of the various treatments on the latency time were expressed as the Hot Plate Analgesia Index -HPAI (Table I)[15].

Drugs Utilised

Morphine hydrochloride and Sodium Dipyrone were used as standards.

A 10 % hydroalcoholic solution was used to dilute the test compound, physiological solution (NaCl 0,9%) was used to dissolve the standard compounds, and aqueous Tween 80 (10%) for hexacarbonyl chromium and pure anisole samples.

The doses for standards and tested compounds were shown in Table I.

Statistical Analysis

A statistical analysis was made using the t-Student and Anova methods for $p<0.05$.

**Results and Discussion**

In our preliminary studies we found that the reaction between $Cr(CO)_6$ and anisole in pure butyl acetate gives a low yield of the desired compound due to decomposition. The substitution of butyl acetate by ethyl acetate, however, leads to incomplete reaction. The use of decalin is not convenient for us, because this solvent can not be easily removed under vacuum and flash chromatography would be necessary[5].

We combined the procedures of Mahaffy and Pauson[2] and of Hudecek and Toma[2,4,5,7,18] and carried out the reaction in a mixture of dibutyl ether with 5% of butyl acetate or of ethyl acetate as coordinating co-solvent, adding the advantages of the Mahaffy procedure (easily isolation) with these of Toma methods ( esters are more eficient as co-solvents then THF), without the inconvenients of pure decalin. The need for special care with the purity of reagents and solvents[2,4,5,7,18], was confirmed by us: the yields are very low when impure solvents are used.

We also used a slow bubling of $N_2$ into the reaction medium, during all the reflux time, for removal of formed CO, because is well known that the reaction between a arene and chromium hexacarbonyl is a equilibrium, and removal of the carbon monoxide is necessary for complete reaction[1].

We used this modified procedure to obtain the $\eta^6$-(anisole)-triscarbonyl- chromium(0) complex used in this study. The same procedure was used for the synthesis of some other $\eta^6$-(arene)-triscarbonyl-chromium(0) complexes (of the arenes: diethylphthalate, methyl benzoate,, mesitylene, toluene, benzene, chlorobenzene)[19] among these the new $\eta^6$-(diethylphthalate)-triscarbonyl-chromium(0) (Toma prepared the methyl analogue[10]. The terephthalate[11] analogue is known).

While various authors have prepared and studied arene chromium complexes, no tests of biological activity were made.

On the other side, it was demonstrated that transition metal containing moieties( such as chromium tricarbonyl), when coordinated to arenes have electron acceptor properties[26,27] and is also known that the analgesic activity of arenes is increased by the presence of electron acceptor groups[20-22,,24,25]. For examples, anisole (this work) is a poor analgesic, while phenacetin (4-acetylamino phenetole) is a powerfull analgesic. Aspirin have a electron acceptor group (COOH) and also, the presence of a halogen (Cl) atom in analgesics increase their potency ,by increase of their efficiency as prostaglandin biosynthesis inhibitors.[23]

These observations suggested to us that $\eta^6$-(anisole)-triscarbonyl-chromium(0) may have some degree of analgesic activity.

We carried out analgesic screening of $\eta^6$-(anisole)-triscarbonyl-chromium(0), using the Hot Plate algesimetric method[14] that demonstrate a supra-spinal response[15]. We screened 30 and 50 mg/Kg doses ,injected through the intraperitoneal pathway.

As already mentioned, the analgesic measurements were continued during 40 minutes and expressed as the protection percent[15]. This short action time was selected, because the toxicology and side effects of administration of $\eta^6$-(anisole)-triscarbonyl-chromium(0) were unknown.

We observed that the both 30 and 50 mg/Kg doses showed a significant analgesic effect when compared with these of the control group, and the standards(Table I).

These results suggests that $\eta^6$-(anisole)-triscarbonyl-chromium(0) have analgesic activity, higher then dipyrone, but not so high then morphine (Figure 1)

We also tested the precursors, anisole and hexacarbonyl chromium, and they seems to be inactive, as expected, since they not possesses electron acceptor substituents.

**FIGURE 1.** PROTECTION PERCENT OF ATC IN RELATION TO THE STANDARDS.

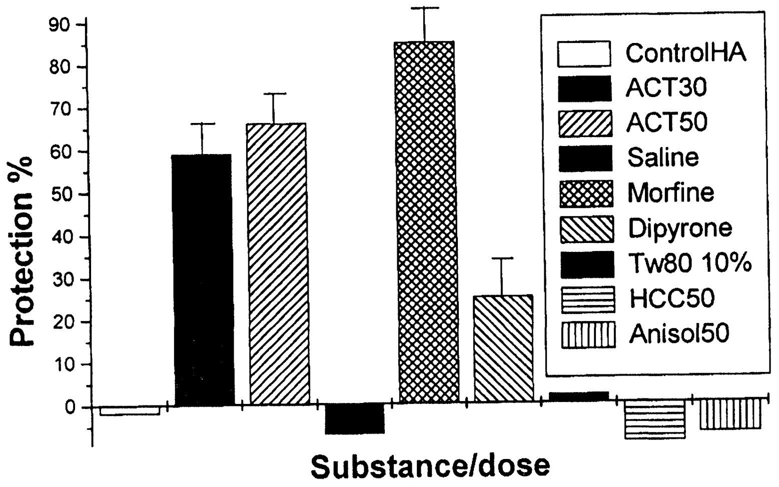

**TABLE I.** ANALGESIC EFFECT OF $\eta^6$-(ANISOLE)-TRISCARBONYL-CHROMIUM(0) (ATC), EXPRESSED AS PROTECTION PERCENT[15]

$$\text{HPAI} = \frac{\text{TEST LATENCY} - \text{BASAL LATENCY}}{{}^{*}30 - \text{BASAL LATENCY}} \times 100$$

| Sample | Dose mg/Kg | Reaction Time (HPAI) | | | | Protection % |
|---|---|---|---|---|---|---|
| | | 10 | 20 | 30 | 40 | |
| Control | - | 03 | -08 | -03 | 00 | -02 |
| ATC** | 30 | 49 | 52 | 67 | 67 | 59* |
| ATC** | 50 | 50 | 65 | 75 | 75 | 66* |
| Saline | - | -02 | -05 | -07 | -12 | -07 |
| Morphine | 04 | 93 | 81 | 100 | 65 | 85 |
| Dipyrone | 60 | 24 | 24 | 13 | 37 | 25 |
| Tw80 10% | - | 00 | 05 | 03 | 00 | 02 |
| HCC*** | 50 | -13 | -06 | -06 | -11 | -09 |
| Anisole | 50 | -10 | 04 | -10 | -12 | -07 |

** ATC is $\eta^6$-(anisole)-triscarbonyl-chromium(III)
*Statistical Significance to $p<0.05$
***Hexacarbonyl Chromium

ContHA is the 10 % hydroalcoholic solution, ATC30 and 50 refers to 30 mg/Kg and 50 mg/Kg doses of $\eta^6$-(anisole)triscarbonyl-chromium (III); saline is standard physiological solution (0.9 %), Morphine and Dipyrone are the standards, HCC50 and Anisole50 refer to Hexacarbonyl chromium 30 mg/Kg and anisole 50 mg/Kg, respectively. Tw80 10% refer to their control group.

**Conclusion**

We report a modified method for the synthesis of $\eta^6$-(anisole)-triscarbonyl-chromium(0) and of some other arene tricarbonyl chromium complexes. Screening shows that this compound exhibits high analgesic effect, the first ever reported for an organo-metallic complex.

**Acknowledgements**

We acknowledge to CAPES for bursary to Márcio Luís Andrade e Silva and to FAPESP and University of Franca for financial support.

**References**

1 - G. K. I. Magomedov, *J. Organomet. Chem.*, **385**, 113 (1989).
2 - M. Hudecek and S. Toma, *J. Organomet. Chem.*, **393**, 115 (1990).
3 - C. A. L. Mahaffy, P. L. Pauson, M. D. Rausch and W. Lee, *Inorg. Syn.*, **19**, 154 (1979).
4 - M. Hudecek and S. Toma, *J. Organomet. Chem.*, **406**, 147 (1991).
5 - M. Prokesova, I. Prokes, M. Hudecek and S. Toma, *Monatsh. Chem.*, **125**, 901 (1994).
6 - B. Nicholls and M. C. Whiting, *J. Chem. Soc.*, 551 (1959).
7 - M. Prokesova and S. Toma, *Chem. Pap.*, **47**, 314, (1993) - C. A. **120**, 323773v (1994).
8 - W. Strohmeier, *Chem. Ber.*, **94**, 2490 (1961).
9 - G. B. Jones and S. B. Heaton, *Tetrahedron: Assymmetry*, **4**, 261 (1993).
10 - S. Toma, Comenius University, Bratislava, Slovakia, personal communication and the generous gift of a sample.
11 - W. Strohmeier and P. Hartmann, *Z. Naturforsch. Teil B*, **18**, 506 (1963).
12 - D. V. Reynolds, *Science*, **164**, 444 - 445, (1969).
13 - R. Melzack and S.C. Dennis, *Raven Press.*, 1 - 26, (1978).
14 - J.M. Besson and A. Chaouch, *Physiol. Rev.*, **67**, 67 - 186, (1987).
15 - R. Melzack, and P. D. Wall, *Science*, **150**, 971 - 979, (1965).
16 - G. Woolfe and A. D. MacDonald, *J. Pharmacol. Exp. Ther.*, **80**, 300 - 307, (1944).
17 -T.L. Yakash; J.C. Yeung and T.A. Rudy, *Brain Res.*, **114**, 83 - 103, (1976).
18 - M. Hudecek, V. Gajda, and S. Toma, *J. Organomet. Chem.*, **413**,155 (1991).
19 - A. Federman Neto, R. N. Bahia, A. D. Lanchote, M. C. S. Duarte, A. P. Vilela and J. Miller, "Improved Synthesis Of Arene-Tricarbonyl Complexes". *Abstracts of XVI International Conference on Organometallic Chemistry*, Sussex, Great-Britain, 200 (1994).
20 - K. Brune, "*New Pharmacological and Epidemiological Data in Analgesics Research*", Birkhauser Verlag, Basel, Switzerland (1990).
21 - A . Korolkovas, "*Fundamentos de Farmacologia Molecular*", Edart, São Paulo, Brazil, 2nd ed., 146,156 (1977).
22 - G. H. Hamor in W. O . Foye, "*Principles of Medicinal Chemistry*", Lea & Febiger, Philadelphia, USA, 2nd ed., 565 (1976).
23 - A . Korolkovas and J. H. Burckhalter, "*Química Farmacêutica*", Guanabara Dois, Rio de Janeiro, Brazil, 212 (1982).
24 - R. E. Willete in J. N. Delgado and W . A . Remers, "*Wilson and Gisvold's Textbook of Organic Medicinal and Pharmaceutical Chemistry*, 9th ed., 657 (1987).
25 - T. J. Holmes Jr. in J. N. Delgado and W . A . Remers, "*Wilson and Gisvold's Textbook of Organic Medicinal and Pharmaceutical Chemistry*, 9th ed., 871 (1987).
26 - P. C. B. Gomes, E. J. S. Vichi, P. J. S. Moran, A. Federman Neto, M. L. Maroso and J. Miller. *Organometallics*, **15**, 3198 (1996).
27 - I. Balas, D. Jhurry, L.Latxague, S. Grelier, Y. Morel, M. Hamdani, N. Ardoin and D. Astruc. *Bull. Soc. Chim. France,* **127**, 401 (1990).

**Received: June 29, 1998 - Accepted: July 10, 1998 -**
**Received in revised camera-ready format: January 19, 1999**

